# Longitudinal Analysis of COVID-19 Patients Shows Age-Associated T Cell Changes Independent of Ongoing Ill-Health

**DOI:** 10.3389/fimmu.2021.676932

**Published:** 2021-05-07

**Authors:** Liam Townsend, Adam H. Dyer, Aifric Naughton, Rachel Kiersey, Dean Holden, Mary Gardiner, Joanne Dowds, Kate O’Brien, Ciaran Bannan, Parthiban Nadarajan, Jean Dunne, Ignacio Martin-Loeches, Padraic G. Fallon, Colm Bergin, Cliona O’Farrelly, Cliona Ni Cheallaigh, Nollaig M. Bourke, Niall Conlon

**Affiliations:** ^1^ Department of Infectious Diseases, St James’s Hospital, Dublin, Ireland; ^2^ Department of Clinical Medicine, School of Medicine, Trinity Translational Medicine Institute, Trinity College Dublin, Dublin, Ireland; ^3^ Department of Medical Gerontology, School of Medicine, Trinity Translational Medicine Institute, Trinity College Dublin, Dublin, Ireland; ^4^ Department of Immunology, St James’s Hospital, Dublin, Ireland; ^5^ Department of Physiotherapy, St James’s Hospital, Dublin, Ireland; ^6^ Department of Respiratory Medicine, St James’s Hospital, Dublin, Ireland; ^7^ Department of Intensive Care Medicine, St James’s Hospital, Dublin, Ireland; ^8^ School of Medicine, Trinity Biomedical Sciences Institute, Trinity College Dublin, Dublin, Ireland; ^9^ School of Biochemistry and Immunology, Trinity Biomedical Sciences Institute, Trinity College Dublin, Dublin, Ireland; ^10^ School of Medicine, Trinity College Dublin, Dublin, Ireland; ^11^ Department of Immunology, School of Medicine, Trinity College, Dublin, Ireland

**Keywords:** COVID-19, immunophenotyping, immune recovery, T cells, fatigue, ageing

## Abstract

**Objectives:**

The immunological and inflammatory changes following acute COVID-19 are hugely variable. Persistent clinical symptoms following resolution of initial infection, termed *long COVID*, are also hugely variable, but association with immunological changes has not been described. We investigate changing immunological parameters in convalescent COVID-19 and interrogate their potential relationships with persistent symptoms.

**Methods:**

We performed paired immunophenotyping at initial SARS-CoV-2 infection and convalescence (n=40, median 68 days) and validated findings in 71 further patients at median 101 days convalescence. Results were compared to 40 pre-pandemic controls. Fatigue and exercise tolerance were assessed as cardinal features of *long COVID* using the Chalder Fatigue Scale and 6-minute-walk test. The relationships between these clinical outcomes and convalescent immunological results were investigated.

**Results:**

We identify persistent expansion of intermediate monocytes, effector CD8+, activated CD4+ and CD8+ T cells, and reduced naïve CD4+ and CD8+ T cells at 68 days, with activated CD8+ T cells remaining increased at 101 days. Patients >60 years also demonstrate reduced naïve CD4+ and CD8+ T cells and expanded activated CD4+ T cells at 101 days. Ill-health, fatigue, and reduced exercise tolerance were common in this cohort. These symptoms were not associated with immune cell populations or circulating inflammatory cytokines.

**Conclusion:**

We demonstrate myeloid recovery but persistent T cell abnormalities in convalescent COVID-19 patients more than three months after initial infection. These changes are more marked with age and are independent of ongoing subjective ill-health, fatigue and reduced exercise tolerance.

**Graphical Abstract:**
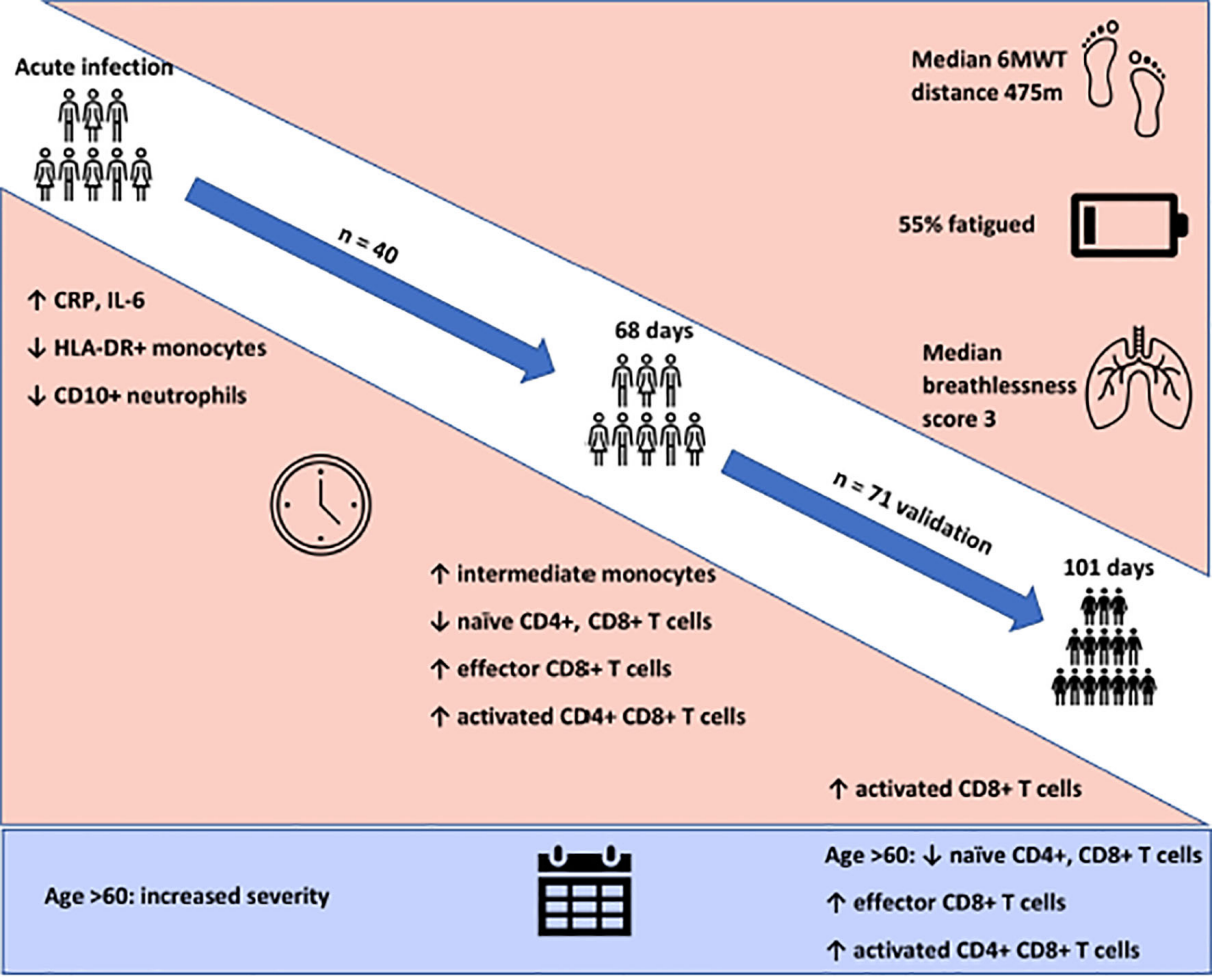


## Introduction

COVID-19, caused by the SARS-CoV-2 virus, is responsible for the largest global pandemic in modern medicine (1). The features of acute illness are well-described, ranging from disturbance in smell and mild coryzal symptoms to acute respiratory failure and the need for invasive mechanical ventilation (2, 3). Age is strongly associated with disease severity, with older individuals suffering poorer outcomes (4, 5). The immunological changes associated with severe disease are also known, with increased inflammatory proteins, coagulopathy and changes in myeloid cell populations reported (6–8). In particular, severe COVID-19 is characterised by expansion of immature myeloid populations, with loss of HLA-DR expression by monocytes and loss of CD10 expression on neutrophils (9, 10). Panlymphopenia is also prominent, with CD4+ T cells particularly affected (11, 12).

In contrast to the well-characterised inflammatory and immunological signature of acute disease, relatively little is known about resolution of inflammatory markers and immune cell population changes during the convalescent period. While fluctuations in immune populations in acute disease have been reported, the medium-term convalescent characteristics remain poorly-described (13). These gaps in current knowledge of convalescence are of immediate importance, with the emergence of prolonged symptoms following resolution of acute infection, termed *long COVID* (14). The immunological features of this syndrome are only being described at present, with short-term follow up (less than one month) of non-hospitalised patients showing expansion of activated CD4+ and CD8+ T lymphocytes (15). Despite the importance of this issue towards understanding the long-term consequences of COVID-19, further insight into the duration of these changes, and the contribution of immune responses to the symptoms of *long COVID*, is lacking.

The clinical characteristics of long COVID are protean and an agreed definition has yet to be found; the most common symptoms include fatigue, shortness of breath and reduced exercise tolerance (16, 17). It has been proposed that post-COVID fatigue and breathlessness are associated with deconditioning, and hypothesised to be due to persistent inflammation or immune activation (18). However, these connections remain unexplored.

Here, we hypothesise that, given the strong association between immune activity and the severity of acute COVID-19 infection, persistent and chronic changes to the immune system may be linked to the long-term effects of COVID-19. We also hypothesise that, given its role in initial disease severity, age may affect immune recovery. Our goal was to determine if persistent inflammatory and immune cell dysregulation was evident in the aftermath of SARS-CoV-2 infection. We further investigated the factors that might be associated with potential persistent immune dysregulation, including age, and to interrogate the relationship between these measures and physical ill-health post-COVID-19.

## Methods

### Study Setting and Participants

This study was carried out in the post-COVID-19 clinic at St James’s Hospital, Dublin, Ireland. Appointments were offered to all individuals with a positive SARS-CoV-2 nasopharyngeal swab PCR at our institution between March and May 2020, including both those hospitalised and those managed in the community during their acute illness. Severity of initial infection was graded as mild (did not require hospitalisation), moderate (required hospitalisation) or severe (required intensive care unit admission). Supplemental oxygen requirements and the presence chest x-ray changes during acute infection were recorded. Appointments were offered at a minimum of six weeks following resolution of symptoms or hospital discharge. Outpatient appointments were not offered to residents in long term care facilities.

### Inflammatory Makers and Immunophenotyping

Blood sampling was incorporated as part of routine phlebotomy occurring on the same day as study participation. An identical sampling and analytic pipeline was implemented on the cohort during acute infection. IL-6, IL-8, IL-1β, TNF-α and soluble CD25 levels were measured in serum by ELISA (Ella ProteinSimple). Immunophenotyping by flow cytometry was carried out on fresh whole EDTA-treated blood and samples were analysed on a FACS Canto II Flow Cytometer (BD San Jose USA), using BD DIVA v8 and FLO Jo v10 software. BD FACSCanto™ clinical software was used for acquisition of BD Multitest™ 6-colour TBNK and TruCount tubes. All other immunophenotyping samples were analysed using BD DIVA v8 and FLO Jo v10 software (**Supplemental Figure 1**). The frequency and absolute cell counts of CD45+ T cells (CD3+, CD4+ and CD8+), B cells (CD19+) and NK cells (CD16+CD56+) were generated by BD Multitest™ 6-colour TBNK and TruCount method. Naive and effector CD4+ or CD8+ T cells were characterised for expression of CD27, CD45RA and CD197. T cell activation was assessed by CD38 and HLA-DR expression. The complete phenotypic identification of lymphocyte subsets were naïve CD4+ (CD3+CD4+CD45RA+CD27+), naïve CD8+ (CD3+CD8+CD45RA+CD27+CD197+), effector CD8+ (CD3+CD4-CD45RA+CD27-CD197-), activated CD4+ (CD3+CD4+HLADR+CD38+) and activated CD8+ (CD3+CD8+HLADR+CD38+). Absolute cell counts for naïve effector and activated T cells were calculated using the absolute frequencies of parent populations acquired from the BD TruCount tubes. Cell phenotyping assays were validated and accredited in line with ISO15189 standards. Classical, intermediate and non-classical monocytes were characterised by CD14 and CD16 expression. The maturation status of CD16+ neutrophils was evaluated by CD10 expression. Antibodies used in flow cytometry phenotyping are in **Supplemental Table 1**. The reference ranges for all assays were generated using a panel of 40 healthy controls and were established in a pre-pandemic setting.

### Physical Health Assessment

Physical health assessment occurred at time of outpatient assessment and convalescent immunophenotyping. Patients were asked a binary question as to whether or not they felt back to full health. Fatigue was assessed using the Chalder Fatigue Scale (CFQ-11) (19, 20). Participants answer eleven questions in relation to physical and psychological fatigue, with reference to the past month in comparison to their pre-COVID-19 baseline. A Likert scale (0-3) is used to measure responses, constructing a total score ranging from 0 to 33 (21).

The CFQ-11 also allows differentiation of “cases” vs “non-cases” where scores 0 and 1 (*Better than usual/No worse than usual*) are scored a zero and scores 2 and 3 (*Worse than usual/Much worse than usual*) are scored a 1 (bimodal scoring). Those with a total score of four or greater are considered to meet the criteria for fatigue. This latter method for *caseness* resembles other fatigue questionnaires (21–24).

To assess cardiopulmonary and musculoskeletal function, a 6MWT was used, with total distance covered recorded (25, 26). The MBS, widely used in both healthy and diseased states to analyse exertion during submaximal exercise, assessed perceived exertion during the 6MWT (range 0 -10) (27, 28).

### Statistical Analysis

All statistical analysis was carried out using STATA v15.0 (Texas, USA) and statistical significance considered p<0.05. Descriptive statistics are reported as means with standard deviations (SD) and median with interquartile ranges (IQR) as appropriate.

Between-group differences were assessed using t-tests, Wilcoxon rank-sum and chi-square tests according to underlying data type and distribution. Paired analysis of laboratory parameters and immune cell populations between matched acute and post COVID samples from the same patients were carried out using Wilcoxon sign-rank test. Linear regression was used to model the relationship between immune cell parameters (independent variable) and CFQ-11/6MWT (distance covered in metres)/MBS score using separate linear models. These were performed unadjusted in the first instance (Model 1), followed by adjustment for age, sex, and severity of initial infection (Model 2). Results are presented as β coefficients with corresponding 95% confidence intervals (Cis) and *p* values. Correlation analysis between parameters was performed using Spearman correlation tests. Bonferroni correction was applied by dividing the alpha level by number of comparisons. Adjusted significance levels are stated in the relevant figure/table legends.

### Study Approval

Ethical approval for the current study was obtained from the Tallaght University Hospital (TUH)/St James’s Hospital (SJH) Joint Research Ethics Committee (reference REC 2020-03). Informed consent was obtained from all participants in the current study in accordance with the Declaration of Helsinki (29).

## Results

### Participant Characteristics

Clinic appointments were offered to 356 patients, of whom 111 (31%) attended (**Supplemental Figure 2**). We recruited three cohorts for this study. Cohort one comprised of forty participants (aged 51.4 ± 16.9 years, 52.5% female) recruited for matched longitudinal blood sampling and immunophenotyping at (i) time of initial COVID-19 disease and (median time from symptom onset to sampling 8 days, IQR 5 – 10) (ii) ten-week follow-up (median: 68, 61-72 days). Of these, 8 had mild (not hospitalised), 24 moderate (hospitalised) and 8 severe (required admission to Intensive Care Unit) acute COVID-19 disease. Supplemental oxygen was required for 27/40 (67.5%) of patients; of these receiving supplemental oxygen, the median fraction of inspired oxygen delivered was 40% (IQR 28 – 60). Chest x-ray changes consistent with SARS-CoV-2 infection were seen in 25/40 (62.5%) of patients, all of whom were hospitalised.

Cohort two comprised of 71 individuals (aged 44.3 ± 14.1 years; 70% female) recruited from the post-COVID clinic at fourteen weeks (median: 101, 76-117 days) after initial COVID-19 illness. Cohort two was younger, had lower levels of frailty and had a greater proportion of females in comparison to cohort one. Cohort two was also predominantly healthcare workers. In cohort two, 47 had mild, 18 had moderate, and 6 had severe acute COVID. All patients had lymphoid and myeloid immunophenotyping and detailed clinical and health assessments performed at outpatient appointment. The combined sample consists of both cohort one and cohort two. Detailed characteristics of cohort one, cohort two and the combined samples are presented in **Table 1**.

**Table 1:** Cohort characteristics.

Characteristic	All (n = 111)	Cohort One (n = 40)	Cohort Two (N = 71)	Statistic
Sex, female, n (%)	70 (63.06)	21 (52.5)	49 (70)	χ^2^ = 8.3, p = 0.004
Mean age (SD)	45.9 (14.9)	51.4 (16.9)	44.3 (14.1)	t = 2.2, p = 0.01
Clinical Frailty Score, median (IQR)	1 (1 – 2)	2 (1 – 2.5)	1 (1-2)	z = -4.3, p <0.001
Co-morbidities, median, n (IQR)	1 (0 – 2)	1 (0 – 2)	0 (0-2)	z = -1.11, p = 0.27
Co-medications, median, n (IQR)	1 (0 – 3)	1 (0 – 4)	1 (0-2)	z = -1.14, p = 0.25
Admission during acute infection, n (%)	48 (43)	30 (75)	18 (25)	χ^2^ = 25.7, p <0.001
Admission to ICU, n (%)	14 (12.6)	8 (20)	6 (8)	χ^2^ = 3.1, p = 0.08
Time to follow up, days, median (IQR)	82 (67 – 112)	68 (60.5 – 71)	101 (76-117)	z = 5.04, p <0.001
Feel back to full health (yes), n (%)	37 (33)	22 (55)	15 (21.2)	χ^2^ = 16.7, p <0.001
Healthcare worker, n (%)	74 (66.7)	15 (37.5)	59 (83)	χ^2^ = 23.9, p <0.001
Distance at 6MWT, m, median (IQR)	475 (415 – 540)	435 (390 – 540)	480 (430-540)	z = 4.5, p = 0.65
MBS, median (IQR)	3 (2 – 5)	3 (2 – 5)	3 (2 - 5)	z = -0.42, p = 0.68
Fatigue score, median (IQR)	15 (11 – 20)	13 (11 – 18)	17 (12 - 21)	z = 1.58, p = 0.11
Fatigue *caseness*, n (%)	61 (55)	15 (37.5)	46 (65)	χ^2^ = 7.7, p 0.01

χ2, Chi-squared test; t, t-test; z, Wilcoxon rank-sum test.6MWT, 6-minute-walk test; MBS, Modified Borg Dyspnoea Scale.

Cohort three comprised of forty healthy pre-pandemic controls (aged 47.3 ± 15.3 years; 55% female) and was used for comparison of extended T cell immunophenotyping parameters, with 20 of these also having myeloid immunophenotyping performed.

The commonest comorbidities in the cohorts were cardiovascular (primarily hypertension; 15% cohort 1, 22% cohort 2, 12.5% controls), respiratory (primarily asthma; 15% cohort 1, 10% cohort 2, 10% cohort 3) and metabolic (primarily type two diabetes; 17.5% cohort 1, 17% cohort 2, 10% controls). In keeping with these comorbidities, the commonest co-medications were anti-hypertensives (cohort 1 14%, cohort 2 21%), lipid-lowering therapies (cohort 1 11%, cohort 2 7%), inhaled respiratory agents (cohort 1 10%, cohort 2 9%) and oral hypoglycaemic agents (cohort 1 4%, cohort 2 6%). One patient in cohort one was taking the tyrosine kinase inhibitor ibrutinib for chronic lymphocytic leukaemia. No other patients in cohort one and no patients in cohort two were receiving immunomodulatory therapy for pre-existing conditions, excluding inhaled corticosteroids. There were no age/sex differences between controls and cohort one (t = 0.9, p = 0.35; χ^2^ = 2.5, p = 0.11 respectively) or cohort two (t = 1.1, p = 0.27; χ^2^ = 0.05, p = 0.82 respectively).

Furthermore, we assessed the use of disease-modifying agents against SARS-CoV-2 in those who underwent immunophenotyping during acute infection. At the point of recruitment, the national guidelines for therapy supported the use of combined hydroxychloroquine (HCQ) (400mg twice daily on day one, then 200mg twice daily for four days) and azithromycin (500mg once daily on day one, then 250mg once daily for two days). Twenty-two patients (22/40, 55%) received this combination, and therapy was administered at a median of 3 days prior to immunophenotyping. Three patients received corticosteroid therapy (dexamethasone in one case and hydrocortisone in the other two) as an adjunct to hydroxychloroquine/azithromycin. In all cases these were administered three days prior to immunophenotyping. No patients received remdesivir or any other targeted therapy. The 22 patients receiving HCQ were all admitted during acute infection, in line with our treatment guidelines. In keeping with this, patients receiving HCQ were older (mean age 55 vs 44, t=-2.18, p=0.04). There were no sex differences between those receiving HCQ and those who did not (χ2 0.4, p=0.53), and there were no differences in numbers of co-morbidities or co-medications between the groups.

### Persistent Ill-Health Evident at 82 Days Following COVID-19

We first investigated the prevalence of the cardinal features of *long COVID* in our cohort. All participants were assessed for ongoing ill-health, fatigue and exercise tolerance at time of convalescent immunophenotyping (median 82 days, IQR 67 – 112). Most patients (71/111, 64%) reported that they did not feel back to full health, while 61 (55%) met the case definition for fatigue. The median fatigue score for the cohort as a whole was 15 (IQR 11 – 20), while the median fatigue score of those who met the case definition for fatigue was 20 (IQR 17 – 23) (**Table 1**). Two-thirds of participants (76/111; 68.4%) underwent a six-minute-walk test (6MWT). The median distance covered was 475m (IQR 415 – 540). The median maximal Modified Borg Dyspnoea Scale (MBS) score reported was 3 (IQR 2 – 5). The median distance covered by convalescent individuals was lower than that seen in healthy populations, but higher than that reported in post-ARDS patients (30, 31). These findings demonstrate that the primary features of *long COVID* are common in our convalescent cohort who attended for follow up appointment. We therefore wanted to further investigate if there were further physiological changes, particularly in relation to immunity, in COVID convalescence.

### Recovery From COVID-19 Is Associated With Resolution of Inflammation, Coagulopathy, and Cell Turnover

**Figure 1:**
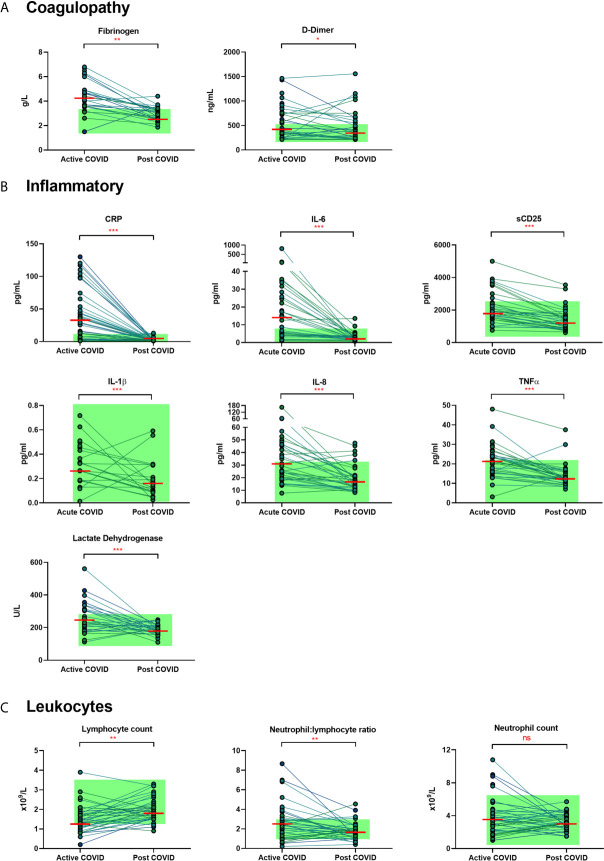
Matched results of n = 40 patients from acute to convalescent (median 68 days). **(A)** Coagulopathy (fibrinogen, D-dimer) **(B)** Inflammatory (CRP, IL-6, sCD25, IL-1β, IL-8, TNFα, LDH) **(C)** Cell turnover (lymphocytes, neutrophil: lymphocyte ratio, neutrophils). Shaded areas show normal ranges for each measure. Differences between paired samples assessed by Wilcoxon signed-rank test. Red horizontal line indicates median *p < 0.05, **p < 0.01, ***p < 0.001, ns, Not Significant.

We examined the levels of inflammatory, cell turnover and coagulation markers, all of which are known to be profoundly disturbed during acute COVID-19. Individuals in cohort one with acute COVID-19 had coagulopathy (with increased D-Dimer and fibrinogen), a marked pro-inflammatory response (elevated CRP, IL-6, TNF-α, IL-8 and IL-1β) and lymphopenia, with an increase in the neutrophil: lymphocyte ratio. All of these parameters had significantly improved by ten weeks (**Table 2** and **Figure 1**) in the majority of individuals. Several participants had persistent lymphopenia (5/40, 12.5%) and elevated D-dimer levels (10/40; 25%) at 10 weeks post infection.

**Table 2:** Active COVID-19 is associated with a significant pro-inflammatory response which normalises following resolution of acute infection.

Laboratory Parameter	Reference Range	Values		Statistic	N (%) Abnormal Results	
		Acute COVID-19	Post COVID-19		Acute COVID-19	Post COVID-19
***Routine Laboratory Markers***						
Neutrophil Count (x10^9^/L)	2-7.5	3.5 (2.3-4.4)	3.15 (2.55-4.05)	z =0.47, p =0.64	5 (13%)	0 (0%)
Lymphocyte Count (x10^9^/L)	1.5-3.5	1.3 (1-1.8)	1.9 (1.55-2.35)	z=-2.85, p = 0.004	24 (60%)	5 (13%)
Neutrophil: Lymphocyte ratio		2.5 (1.3-3.9)	1.7 (1.3-2.1)	z =2.82, p = 0.005		
Lactate Dehydrogenase (U/L)	135-250	230 (190-308)	187.5 (165-210)	z = 3.27, p = 0.001	14 (35%)	0 (0%)
Fibrinogen (g/L)	1.9-3.5	4.4 (3.6-6)	3 (2.6-3.4)	z = 2.86, p = 0.004	22 (55%)	2 (5%)
D-Dimer (ng/mL)	0-500	496 (258-851)	396 (215-599)	z = 1.93, p = 0.05	20 (50%)	10 (25%)
C Reactive Protein (mg/L)	0-5	28.15 (3.16-53.58)	1.43 (1.00-1.99)	z = 4.75, p<0.001	31 (78%)	3 (8%)
***Pro-Inflammatory Cytokines***						
IL-6 (pg/mL)		15.3 (4.19-31.7)	2.17 (1.45-3.35)	z = 4.72, p<0.001	22 (55%)	3 (8%)
IL-1β (pg/mL)		0.26 (0.18-0.45)	0.19 (0.11-0.31)	z = 2,1, p = 0.035	1 (2.5%)	1 (2.5%)
TNFα (pg/mL)		21.3 (16-25)	12.2 (10.3-14.8)	z = 3.95, p<0.001	16 (40%)	2 (5%)
IL-8 (pg/mL)		31.1 (20.3-46.4)	16.5 (12.3-21.4)	z = 4.23, p<0.001	17 (42.5%)	4 (10%)
Soluble CD25 (pg/mL)		1,898 (1520-2455)	1,187 (878-1634)	z = 4.61, p<0.001	11 (28%)	2 (5%)

Data presented as medians with IQRs. z, Wilcoxon matched pair.

### Persistent Expansion of Intermediate Monocytes at 10 Weeks Post Infection

**Figure 2:**
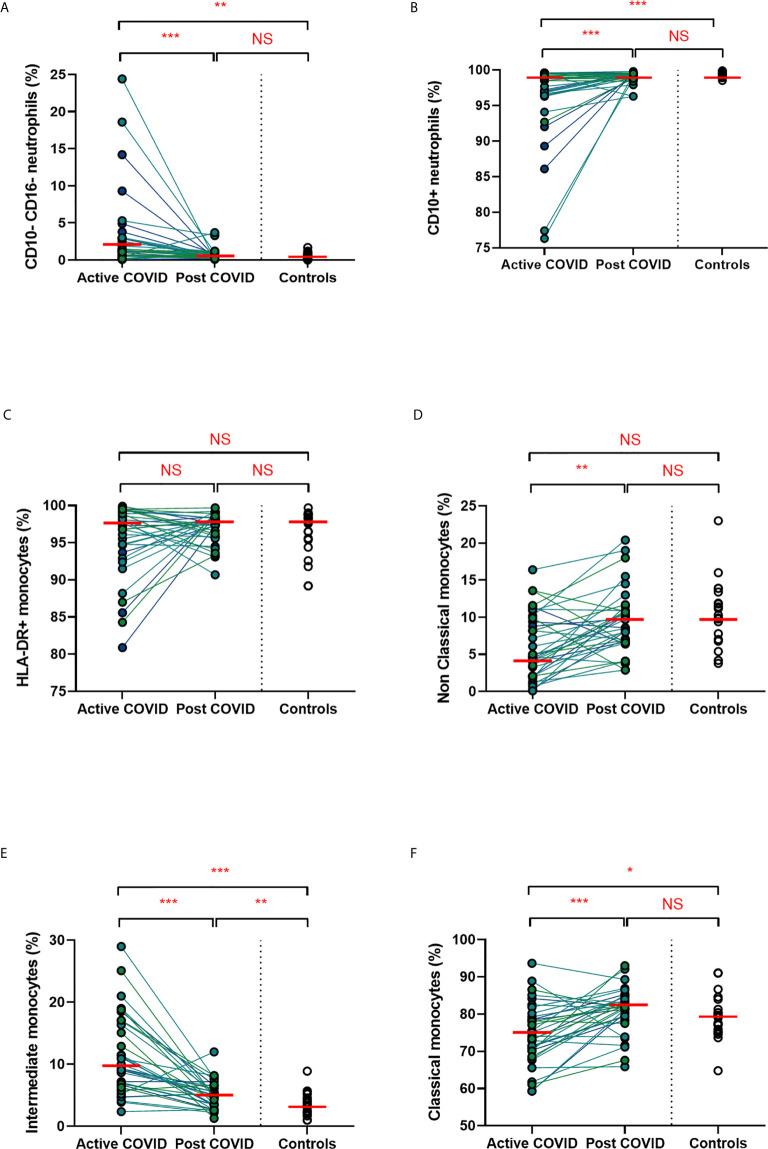
Myeloid populations from acute to convalescent COVID-19. Matched peripheral whole blood myeloid cell proportions from n=40 patients recovered from COVID-19 versus uninfected controls (n=20). **(A)** CD10-CD16- immature neutrophils **(B)** CD10+ neutrophils **(C)** HLA-DR+ monocytes **(D)** non-classical monocytes **(E)** intermediate monocytes **(F)** classical monocytes. Differences between unpaired samples (controls and acute/post COVID) assessed using Wilcoxon rank-sum. Differences between paired samples (acute and post COVID) assessed using Wilcoxon sign-rank tests. Red horizontal line indicates median *p < 0.05, **p < 0.01, ***p < 0.001, NS, Not Significant.

We found that acute COVD-19 was associated with expansion in immature (CD10-CD16-) neutrophils and reduced overall CD10-expressing neutrophils (**Figures 2A, B**). These changes in neutrophils had resolved by ten weeks to a level comparable to that of healthy controls. While HLA-DR+ monocytes increased at convalescence to levels of healthy controls, this increase was not statistically significant; this likely reflects the mixed severities of the populations (**Figure 2C**). We also noted a significant expansion in the proportion of HLA-DR+CD14+CD16+ intermediate monocytes in acute infection, with levels remaining significantly elevated at convalescence in comparison to controls (**Figure 2E**). Changes in the proportion of other monocyte subsets resolved to a level comparable to control participants (**Figures 2D, F**). We found no association between convalescent intermediate monocytes and severity of initial infection (χ^2 =^ 0.58, p=0.76).

### Persistent Changes to T Cells in COVID-19 Convalescence

**Figure 3:**
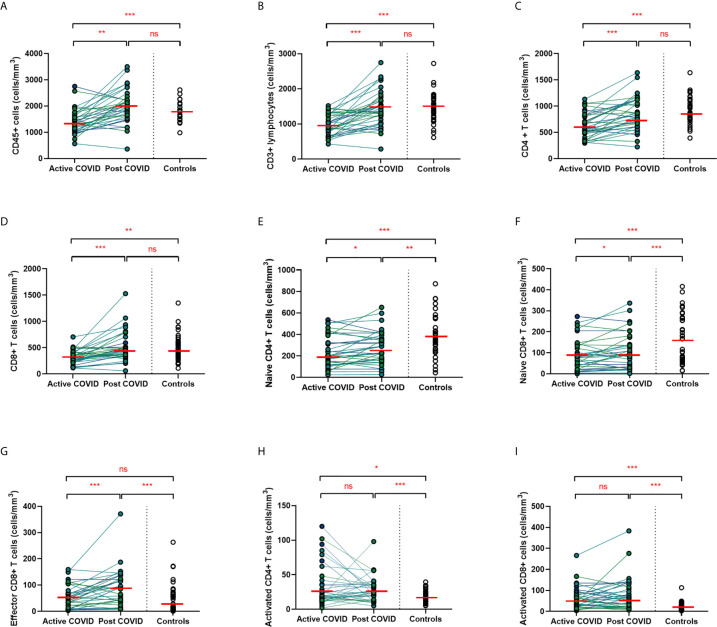
Lymphocyte subsets from acute to convalescent COVID-19. Matched peripheral whole blood lymphoid cell counts from n=40 patients recovered from COVID-19 versus uninfected controls (n=40). **(A)** CD45+ immune cells **(B)** CD3+ lymphocytes **(C)** CD4+ T cells **(D)** CD8+ T cells **(E)** naïve CD4+ T cells **(F)** naïve CD8+ T cells **(G)** effector CD8+ T cells **(H)** activated CD4+ T cells **(I)** activated CD8+ T cells. Differences between unpaired samples (controls and acute/post COVID) assessed using Wilcoxon rank-sum. Differences between paired samples (acute and post COVID) assessed using Wilcoxon sign-rank tests. Red horizontal line indicates median *p < 0.05, **p < 0.01, ***p < 0.001, ns, Not Significant.

A hallmark of acute COVID-19 is profound lymphopenia, so we sought to investigate T cell phenotypes in our longitudinal acute-convalescent cohort. As expected, acute SARS-CoV-2 infection was associated with striking lymphopenia (**Figure 3B**). We found a significant reduction in the total number of CD45+ leukocytes, total lymphocytes (CD3+), CD4+ T cells and CD8+ T cells in comparison to healthy controls (n=40). Despite this significant lymphopenia during acute COVID-19 infection, at ten weeks post-COVID all of these counts had significantly recovered and were similar to those of control participants (**Figures 3A–D**).

On more detailed T cell immunophenotyping, we found that acute infection was associated with a significant reduction in absolute naïve CD4+ (CD3+CD4+CD45RA+CD27+) and CD8+ (CD3+CD8+CD27+CD45RA+CD197+) T cell counts and increased activated CD4+ and CD8+ T cell numbers in comparison to controls. Over the course of ten weeks, whilst naïve CD4+ and CD8+ cell counts had partially recovered, they remained significantly lower than the levels of healthy controls (**Figures 3E, F**). Numbers of effector (CD3+CD4-CD45RA+CD27-CD197-) CD8+ T cells remained expanded over the course of COVID-19 recovery and counts were significantly greater than healthy controls at ten-week follow-up (**Figure 3G**). One of the most notable differences was that activated T cell numbers did not significantly change with resolution of illness; there were significantly higher numbers of activated CD4+ (CD3+ CD4+HLADR+CD38+) and CD8+ (CD3+ CD8+HLADR+CD38+) T cells in patients ten weeks post-COVID compared to healthy controls (**Figures 3H, I**). Interestingly, there was no association between convalescent lymphocyte subset counts and severity of initial infection when comparing those managed as an outpatient, at ward level, and in ICU (naïve CD4+ T cells χ^2 =^ 0.31, p=0.86, naïve CD8+ T cells χ^2 =^ 4.12, p=0.13, effector CD8+ T cells χ^2 =^ 0.29, p=0.19, activated CD4+ T cells χ^2 =^ 0.28, p=0.87, activated CD8+ T cells χ^2 =^ 0.29, p=0.07). However, when comparing hospitalised and non-hospitalised patients with healthy controls, the most marked convalescent T cell subset differences were seen with more severe initial disease (**Supplemental Figure 3**).

Alterations in the B cell compartment were relatively minor, with B cell counts recovering entirely at convalescence (**Supplemental Figure 4A**). Similarly, while acute COVID-19 was also associated with a significant decrease in NK cells, these had resolved to levels of healthy controls by ten weeks after acute infection (**Supplemental Figure 4B**). Collectively, longitudinal follow up of cohort one during convalescence revealed that while the overall panlymphopenia resolved, infected individuals maintained aberrations in certain lymphocyte subset compartments; specifically, persistently reduced naïve CD4+ and CD8+ T cells, in addition to expanded effector CD8+ T cells and activated CD4+ and CD8+ T cells at a median of 68 days following infection. The effect that a short 5-day course of hydroxychloroquine would have on circulating immune cells 68 days after treatment is uncertain. Nevertheless, we investigated between-group differences (HCQ and no HCQ) in the significant results found at convalescence. Wilcoxon rank-sum test was used to assess differences between those who received HCQ and those who did not in the convalescent cell populations noted to be significantly different at 68 days. No differences were seen in intermediate monocytes (z=-0.60, p=0.55), naïve CD4+ cells (z=-0.14, p=0.89), naïve CD8+ cells (z=1.14, p=0.26), effector CD8+ cells (z=-1.56, p=0.12), activated CD4+ cells (z=-0.55, p=0.58), or activated CD8+ cells (z=-1.95, p=0.052).

**Figure 4:**
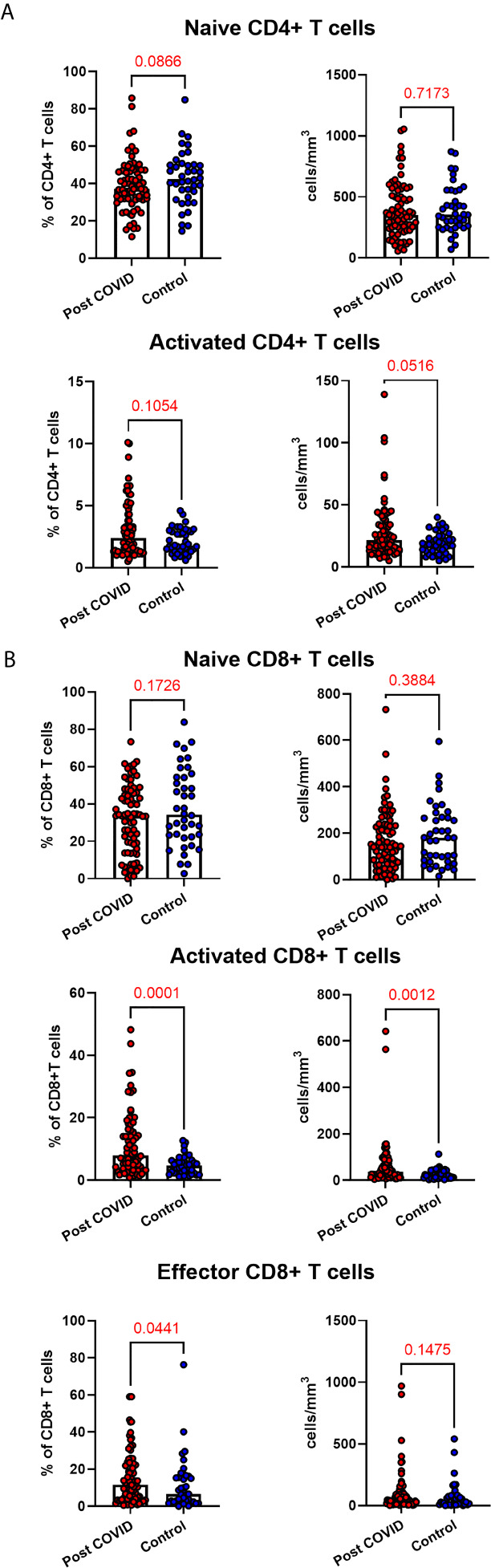
Lymphocyte subsets in convalescent COVID-19. Convalescent lymphocyte subsets from n=71 patients recovered from COVID-19 versus n=40 uninfected controls. **(A)** CD4+ T cells, showing proportion and absolute number of naïve and activated CD4+ T cells **(B)** CD8+ T cells, showing proportion and absolute number of naïve, activated and effector CD8+ T cells, Wilcoxon rank-sum test used to assess differences.

Given the persistent T-cell subset changes seen in cohort one, as well as the expansion in intermediate monocytes, we sought to validate these changes in a larger cohort of convalescent individuals with blood samples obtained at a longer follow time of 101 days (cohort two). The observations in cohort one were confirmed, with no differences in numbers of total CD45+ leukocytes, total lymphocytes (CD3+), CD4+ T cells or CD8+ T cells at 14 weeks in comparison to controls (**Supplemental Figure 5**). In contrast to the paired analysis at 10 weeks following acute infection, we observed no significant differences in naïve CD4+ or CD8+ cells, suggesting these cells had resolved to normal levels by 14 weeks in convalescence (**Figure 4**). However, the increased numbers and proportions of activated CD8+ T cells in post-COVID samples persisted, even at this longer time point of follow up in comparison to cohort one (median 101 days versus 68 days) **(** **Figure 4** **)**. There were no changes in activated CD4+ T cells seen, while convalescent COVID patients had an increased proportion of effector CD8+ T cells. We also stratified these lymphocyte changes by severity of initial infection, comparing patients admitted and non-admitted during initial infection with controls. This demonstrated both disease-related and severity-related effects on lymphocyte populations (**Supplemental Figure 6**). Again, similar to the results from cohort one, the most marked T cell subset differences were seen in those with more severe initial infection. The significant differences in proportion of intermediate monocytes were not seen in this cohort, with all monocyte populations returning to levels similar to controls (**Supplemental Figure 7**).

### Lymphocyte Subset Changes in Convalescent COVID-19 Are Associated With Increasing Age

**Figure 5:**
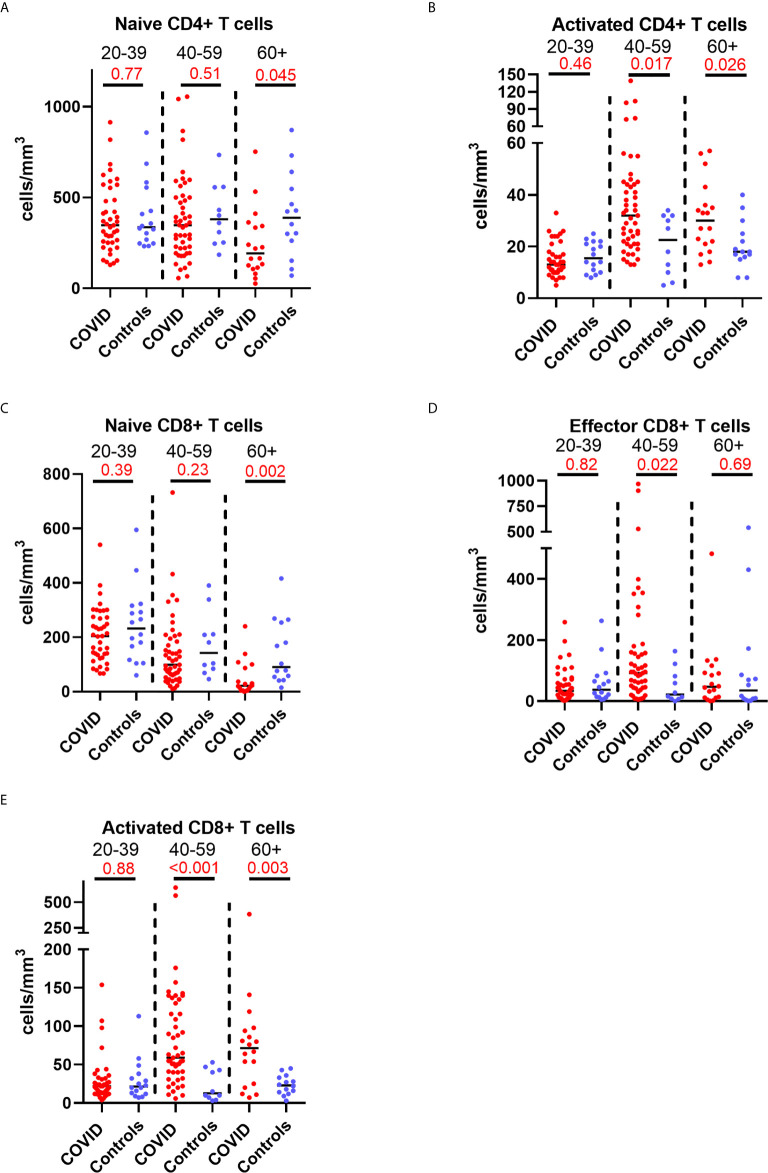
Age-associated changes in convalescent lymphocyte subsets versus age-matched controls. Lymphocyte immunophenotyping of convalescent COVID patients (n=111) broken down by age with age-matched controls, showing absolute number of **(A)** naïve CD4+ T cells and **(B)** activated CD4+ T cells, and **(C)** naïve CD8+ T cells, **(D)** activated CD8+ T cells and **(E)** effector CD8+ T cells. Differences assessed by Wilcoxon rank-sum test. Bonferroni correction, significance p<0.02.

Older patients have worse outcomes in acute SARS-CoV-2 infection, but little is known regarding recovery of their immune system following infection. As we had noted interesting lymphocyte abnormalities post-COVID, we further investigated if age influences any of the dynamic changes T cell responses seen in convalescence. We stratified our convalescent patients into 20-year age brackets: 20 – 39 (n=43), 40 – 59 (n=49) and 60 – 80 (n=19). There was no difference in time to follow up between the three age groups (z=1.5, p=0.47). The older 60 – 80 cohort were more likely to have been admitted during acute infection than the youngest (z=4.62, p=<0.001) and 40 – 59 (z=2.77, p=0.02) cohorts. We also stratified our healthy cohort into identical age brackets: 20 – 39 (n=16), 40 – 59 (n=10), and 60 – 80 (n=14). There were distinct age-associated changes seen in lymphoid populations in the convalescent cohort, with an age-associated decline in both absolute number and proportions of naïve CD4+ and CD8+ cells and an age-associated increase in activated CD4+ and CD8+ cells (**Supplemental Figure 8**). There were minor increases in effector CD8+ T cells with age (**Supplemental Figure 8**). We also investigated disease-associated effects by comparing these results to age-matched controls. There were distinct differences noted, with the most dramatic being in the oldest cohort, with post-COVID individuals having reduced number of naïve CD4+ and naïve CD8+ (**Figures 5A, C**) and increased number and proportion of activated CD4+ and CD8+ cells (**Figures 5B, E**) compared to age-matched controls. There were no differences between the age-matched controls and infected individuals in the youngest cohort. The 40 – 59 age group showed increased number of effector CD8+ T cells (**Figure 5D**), activated CD4+ and activated CD8+ T cells in comparison to uninfected controls, with no differences in naïve T cell counts. These changes were mirrored in the proportion of T cell populations across age groups (**Supplemental Figure 9**). These data suggest that in those >60 years of age the post-COVID changes in T cells persist longer than in younger individuals.

Given the earlier evidence that severity of initial infection impacts on convalescent T cell subsets, we wanted to investigate whether the age-associated effect seen in our data was independent of these severity effects. We built a multivariable linear regression model, including the convalescent results of all 111 patients (cohort 1 and cohort 2 combined) as well as the results of the n=40 controls. We assessed the relationship of the five T lymphocyte subsets (naïve and activated CD4+ T cells, effector CD8+ T cells, and naïve and activated CD8+ T cells) with both age and severity, and also included sex as a variable. We classed severity as requiring admission during initial infection, mirroring the classification used previously. Both age and severity of infection are independently associated with the changes in activated CD4+ cell proportions, as well as proportions of naïve, effector and activated CD8+ cells. The results of this analysis are shown in **Table 3**. There was no significant effect seen with sex and any subpopulation, so these results have not been presented.

**Table 3:** Relationship between T cell subsets, age and severity in n=40 controls and n=111 convalescent COVID-19 patients.

	Naïve CD4 Percentage		Naïve CD4 Count	
	β coefficient (95% CI)	p value	β coefficient (95% CI)	p value
**Age**	-0.16 (-0.32 – -0.002)	0.048	-1.8 (-4.1 – 0.6)	0.15
**Disease status** **Control** **Not admitted** **Admitted**				
	Reference -4.2 (-9.7 – 1.4) -9.9 (-15.8 - -4.1)	n/a 0.14 0.001	Reference -5.6 (-87.8 – 76.5) -106.4 (-192.5 – -20.3)	n/a 0.89 0.02
	**Activated CD4 Percentage**		**Activated CD4 Count**	
	β coefficient (95% CI)	p value	β coefficient (95% CI)	p value
**Age**	0.06 (0.04 – 0.08)	<0.0001	0.44 (0.22 – 0.66)	<0.0001
**Disease status** **Control** **Not admitted** **Admitted**				
	Reference 1.1 (0.4 – 1.7) 1.2 (0.5 – 1.9)	n/a 0.002 0.001	Reference 12.4 (4.8 – 20.0) 7.9 (-0.01 – 15.8)	n/a 0.001 0.05
	**Effector CD8 Percentage**		**Effector CD8 Count**	
	β coefficient (95% CI)	p value	β coefficient (95% CI)	p value
**Age**	0.28 (0.12 – 0.44)	0.001	1.4 (-0.4 – 3.3)	0.12
**Disease status** **Control** **Not admitted** **Admitted**				
	Reference 4.6 (-0.9 – 10.2) 7.8 (1.9 – 13.6)	n/a 0.10 0.01	Reference 25.7 (-37.5 – 88.9) 59.6 (-6.6 – 125.9)	n/a 0.42 0.08
	**Naive CD8 Percentage**		**Naive CD8 Count**	
	β coefficient (95% CI)	p value	β coefficient (95% CI)	p value
**Age**	-0.5 (-0.7 – -0.3)	<0.0001	-3.5 (-4.8 – -2.2)	<0.0001
**Disease status** **Control** **Not admitted** **Admitted**				
	Reference -6.1 (-12.8 - -.5) -15.7 (-22.7 – -8.7)	n/a 0.07 <0.0001	Reference -29.9 (-71.6 – 17.7) -72.5 (-119.3 – -25.7)	n/a 0.24 0.003
	**Activated CD8 Percentage**		**Activated CD8 Count**	
	β coefficient (95% CI)	p value	β coefficient (95% CI)	p value
**Age**	0.27 (0.17 – 0.37)	<0.0001	1.0 (0.1 – 2.0)	0.03
**Disease status** **Control** **Not admitted** **Admitted**				
	Reference 6.6 (3.3 – 10.0) 10.5 (7.0 – 14.0)	n/a <0.0001 <0.0001	Reference 37.0 (4.7 – 69.4) 55.7 (21.8 – 89.6)	n/a 0.03 0.001

Multivariable linear regression, adjusted for disease status, age, and sex. CI, confidence interval.

### Persistent Symptoms Are Independent of T Cell Immunophenotype Following COVID-19 Illness

**Figure 6:**
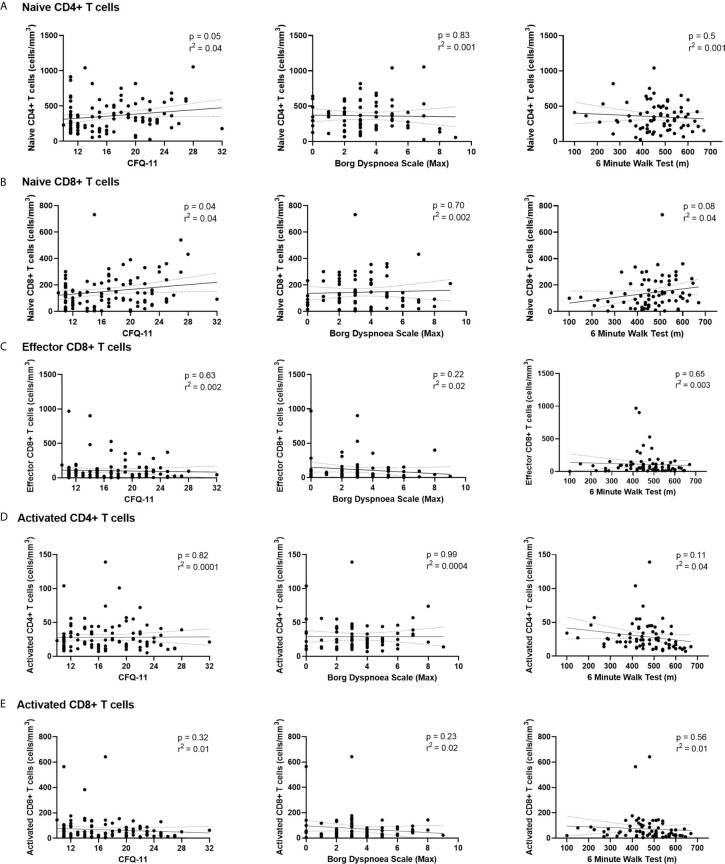
Relationships of lymphocyte subsets with physical health measures and D-dimers. Relationship with fatigue, perceived exertion and 6MWT distance with **(A)** naïve CD4+ T cells, **(B)** naïve CD8+ T cells, **(C)** effector CD8+ T cells, **(D)** activated CD4+ T cells and **(E)** activated CD8+ T cells. Relationships assessed using linear regression. CFQ-11, Chalder Fatigue Questionnaire-11.

We have demonstrated the high prevalence of ill-health and the cardinal features of *long COVID* in our cohort Our analysis has also revealed persistent changes to T cells, most notably activated CD8+ T cells, in the convalescent period following COVID-19. We finally wanted to investigate the relationship between immunophenotyping parameters and subjective symptoms of fatigue, as assessed by CFQ-11 score, and exercise tolerance, as assessed by performance on the 6MWT. We used linear regression under unadjusted conditions and controlled for age, sex and clinical frailty score in order to determine relationships. There were no associations between naïve CD4+, naïve CD8+, effector CD8+, activated CD4+, or activated CD8+ T cells and fatigue score, distance reached on the 6MWT or maximal MBS reported under any of the conditions examined (**Table 4** and **Figure 6**). We also evaluated the association between physical health measures and classical, intermediate and non-classical monocyte populations, given the intermediate monocyte differences we saw at 68 days. There were no associations with monocyte populations (**Supplemental Figure 10**).

**Table 4:** No relationship between T cell subsets and symptoms of fatigue, dyspnoea or distance walked on the six-minute walk test post-COVID-19.

	CFQ-11				6MWT (meters)				Borg Dyspnoea Scale			
	Model 1		Model 2 (adj.)		Model 1		Model 2 (adj.)		Model 1		Model 2 (adj.)		
	β Coeff (95% CI)	P	Adj. β Coeff (95% CI)	p	β Coeff (95% CI)	p	Adj. β Coeff (95% CI)	p	β Coeff (95% CI)	p	Adj. β Coeff (95% CI)	p
**Naïve CD4 Count**	0.005 (0.00, 0.01)	0.046	0.004 (-0.001, 0.01)	0.07	-0.05 (-0.19, 0.09)	0.50	-0.14 (-0.26, -0.02)	0.02	-0.0002 (-0.003, 0.002)	0.83	-0.0002 (-0.003, 0.002)	0.89
**Naïve CD8 Count**	0.01 (0.001, 0.02)	0.04	0.01 (0.001, 0.02)	0.04	0.20 (-0.03, 0.42)	0.08	-0.10 (-0.34, 0.13)	0.39	0.001 (-0.003, 0.005)	0.70	0.003 (-0.002, 0.008)	0.21
**Effector CD8 Count**	-0.002 (-0.01, 0.01)	0.63	0.003 (-0.05, 0.05)	0.92	-0.04 (-0.20, 0.12)	0.65	0.01 (-0.13, 0.15)	0.85	-0.002 (-0.005, 0.001)	0.22	-0.002 (-0.004, 0.001)	0.23
**Activated CD4 Count**	0.04 (-0.69, 0.77)	0.82	-0.01 (-0.06, 0.04)	0.75	-1.04 (-2.31, 0.23)	0.11	-0.0002 (-1.19, 1.19)	0.99	-0.0001 (-0.02, 0.02)	0.99	-0.01 (-0.03, 0.02)	0.48
**Activated CD8 Count**	-0.01 (-0.02, 0.01)	0.32	-0.01 (-0.02, 0.004)	0.20	-0.08 (-0.35, 0.19)	0.56	0.09 (-0.16, 0.33)	0.48	-0.003 (-0.008, 0.002)	0.23	-0.004 (-0.01, 0.001)	0.10

Linear regression. CI, confidence interval. Model 1, unadjusted; Model 2, adjusted for age, sex, Clinical Frailty Scale and severity of acute COVID-19 illness. Bonferroni correction, statistical significance p < 0.01.

These findings suggest that persistent ill-health and fatigue are frequently reported at a medium-term interval following COVID-19, but they are independent of any persistent changes to the immune parameters investigated in this study.

## Discussion

We show evidence of persistent abnormalities in T cell populations following acute SARS-CoV-2 infection that are unrelated to commonly-reported post-COVID symptoms of ill-health. We included the full spectrum of disease, with severity ranging from mild disease managed in the community to requirement for ICU care, in order to capture the complete range of immune recovery. Specifically, we demonstrate an expansion of effector CD8+ T cells, activated CD4+ and CD8+ T cell populations and reduction in naïve CD4+ and CD8+ T cells at ten weeks following acute infection. Further analysis of COVID convalescence found that the expansion of activated CD8+ T lymphocytes is still evident at a median of 101 days following infection. We demonstrate that these changes are more marked in those with more severe initial disease. Equally notable is the recovery post-COVID of myeloid populations to levels similar to healthy controls. While we report persistent expansion of intermediate monocytes at 68 days, all monocyte populations have normalized at 101 days. Given that the myeloid compartment is significantly altered in acute infection, it is reassuring to see resolution of these changes at convalescence. Similarly, routine clinical measures of inflammation and coagulation also return to normal levels. The exception to this is D-dimers, which remain elevated in 18% of patients. Persistent ill-health is common in our cohort, with almost two-thirds (64%) not feeling back to full health, and fatigue seen in more than half the cohort. These are the cardinal features described in *long COVID*. However, these findings of persistent ill-health are independent of the observed changes in immune populations and circulating inflammatory markers.

While persistent expansion of activated T lymphocytes are described in the setting of chronic viral infection, they are less commonly seen following infection by an acute pathogen (32). However, they have been reported in the setting of parvovirus B19 infection, as well as the aftermath of severe influenza A H7N9 infection (33, 34). One of the most striking findings was that these T cell-specific changes are most marked in older >60-year-old participants, with persistent abnormalities across naïve and activated CD4+ and CD8+ T cells, while those aged <40 show complete resolution of these changes. While age is known to affect immune recovery in treated chronic infections such as HIV, there is relatively little known about ageing and immune recovery following acute viral infections (35). The ageing immune system has been implicated in the ageing-associated mortality rate in acute COVID-19 (36, 37). T cell responses are crucial in modulating the immune response and preventing host damage in acute viral infections such as influenza (38).

Impairment of the adaptive immune system with age, in particular immunosenescence, has been well-described (39). Impaired cytotoxic CD8+ T cell responses have been reported in older patients during acute SARS-CoV-2 infection (40). The ability to mount a coordinated immune response to acute SARS-CoV-2 infection declines with age (41, 42). A recent study found there was a general decline in recognition of viral-associated peptides between the ages of 42 and 58 in a pre-pandemic cohort (43). Furthermore, there are poor cross-reactive T cell responses to human coronaviruses seen in older adults (44). This is of particular importance when considering the impact of lasting immunity in previously infected older individuals and how little is known about adaptive immune responses in this population in the context of COVID-19. Another important consideration regarding our results is how such alterations may impact on the potential long-term efficacy of vaccination of older cohorts, where responses have previously been shown to be highly variable (45, 46). These results emphasize the importance of further study into activation and resolution of immune responses in SARS-CoV-2 infection in older individuals, as well as the functional impact of these T cell subpopulation changes.

The strong independent effects of both age and severity of initial infection on convalescent T lymphocyte populations in COVID-19 is novel. It has been noted in prior viral respiratory infections there is persistent expansion of effector T cells following resolution of infection (47). However, these responses have been shown to decline with age (48). These persistent CD8+ lymphocyte changes have also been seen in the convalescent period following Influenza infection, and are associated with severity of initial infection (49). Our data supports the double-hit effect of both advancing age and disease severity on convalescent immune populations following SARS-CoV-2 infection that has seen in other respiratory viral infections.

Interestingly, we found the cardinal features of *long COVID*, namely the presence of fatigue and reduced exercise tolerance, had no association with lymphocyte subset changes. While it is likely that the proportion of those reporting persistent ill-health is enriched in the population choosing to attend follow up clinics, this data highlights ongoing morbidity evidenced by a large burden of fatigue, breathlessness, and ill-health in this cohort. This is reflective of previous clinical descriptors of *long COVID* cohorts (50, 51). Our group have previously reported a high prevalence of post-COVID fatigue, which was independent of severity of initial infection (52). These findings have been replicated in subsequent studies showing persistent ill-health in young, otherwise healthy individuals (53, 54). Our findings further highlight the difficulty in establishing a reliable biomarker for ongoing ill-health following COVID-19 infection.

It will be illuminating to follow the prevalence of autoimmune disease in the general population in the post-pandemic era. This is particularly relevant given the concept of bystander T cell activation during viral infection. Bystander T cell recruitment has been described in both hepatitis A and influenza A infection (55, 56). While the activation of such T cells are considered beneficial in the control of acute infection and protective immunity, such non-specific immune activation has been associated with immunopathology, or host damage (57). Indeed, activation of both CD4+ and CD8+ lymphocytes in acute infection has been associated with the development of a wide array of autoimmune conditions (58, 59). The potential for SARS-CoV-2 to cause immunopathology has been proposed as a mechanism behind multisystem inflammatory syndrome in children (MIS-C), a new paediatric inflammatory condition associated with COVID-19 (60). Autoantibodies against multiple cell types have been demonstrated in this condition, while immune complex formation has also been implicated (61, 62). Furthermore, this is similar to the immunopathology seen in other post-infectious autoimmune conditions, such as Kawasaki disease (63). Pro-thrombotic autoantibody generation has also been described in acute COVID-19 in adult populations, which would link both activated T lymphocytes and elevated D-dimers (64). D-dimer levels have also been reported to correlate with CD8+ lymphocytes in MIS-C (65). Our findings echo previous reports of elevated D-dimers during COVID convalescence and demonstrate concurrent immune perturbations (66, 67). We demonstrate cell population changes that mimic these conditions. However, further characterisation of these persistently activated cells is warranted to inform assessment of their functional consequences.

Our study has several limitations worth noting. It is a single-centre study at a single medium-term interval. However, we have two separate convalescent time points, in addition to data from acute illness. This allows a disease and recovery trajectory to be plotted. We have loss to follow-up, with 31% of patients attending their outpatient appointments. This is a common challenge seen in research conducted in clinical ambulatory care settings. However, our cohort may have an increased burden of symptoms following COVID-19 than that seen in the entire affected population. Our study also does not provide direct functional assessment of the immune cell subsets at a cellular level. However, it provides a detailed description of immune cell recovery, as well as an assessment of circulating inflammatory cytokines. We also did not perform a comprehensive assessment of patient clinical function and recovery post-COVID, but instead focused on the assessment of two cardinal features of *long COVID*, namely fatigue and reduced exercise tolerance.

The results reported here provide insights into the immune consequences of SARS-CoV-2 infection, as well as the age effects on immune recovery. It provides possible mechanisms for immunopathology and should inform the design of ongoing studies into the immunological consequences of COVID-19 and associations with long COVID clinical features.

In conclusion, we report several key findings that add significant knowledge regarding resolution of immunological responses in the convalescent period of COVID-19 infection. Encouragingly, our matched longitudinal patient data shows that all cell counts return towards normal. Although lymphocyte and monocyte abnormalities persist in some individuals up to 68 days, these had resolved by 101 days post infection with the exception of a persistent expansion of activated CD8+ T cells. We show that age, while being strongly associated with poor outcome in acute COVID, is also strongly associated with impaired immunological recovery in convalescence. We also demonstrate that this ageing effect is independent of severity of initial infection. While we demonstrate the burden of persistent ill-health following SARS-CoV-2 infection, this was not associated with any of the immunological changes described here.

## Data Availability Statement

The raw data supporting the conclusions of this article will be made available by the authors, without undue reservation.

## Ethics Statement

The studies involving human participants were reviewed and approved by Tallaght University Hospital (TUH)/St James’s Hospital (SJH) Joint Research Ethics Committee. The patients/participants provided their written informed consent to participate in this study.

## Author Contributions

Conceptualisation: LT, AD, CO’F, CN, and NB. Methodology: LT, AD, AN, RK, DH, MG, JDo, KO’B, PF, NB and NC. Formal Analysis: LT, AD, JDu and NB. Investigation: LT, AN, RK, DH, MG, JDo, KO’B, CBa, PN, IM-L. Resources: JDu, IM-L, CBe, CO’F, CN, NB and NC. Data Curation: LT, AD, AN, RK, JDu and NC. Writing-Original Draft: LT, AD, CN, NB and NC. Writing-Review and Editing: AN, JDu, IM-L, PF, CBe, CO’F, CN, NB and NC. Visualisation: LT, AD, AN, RK, DH, MG and NB. Supervision: IM-L, CBe, CO’F, CNC, NB and NC. Funding Acquisition: LT and NC. All authors contributed to the article and approved the submitted version.

## Funding

LT has been awarded the Irish Clinical Academic Training (ICAT) Programme, supported by the Wellcome Trust and the Health Research Board (Grant Number 203930/B/16/Z), the Health Service Executive, National Doctors Training and Planning and the Health and Social Care, Research and Development Division, Northern Ireland (https://icatprogramme.org/). NC, CN and CO’F are part-funded by a Science Foundation Ireland (SFI) grant, Grant Code 20/SPP/3685. The funders had no role in study design, data collection and analysis, decision to publish, or preparation of the manuscript.

## Conflict of Interest

The authors declare that the research was conducted in the absence of any commercial or financial relationships that could be construed as a potential conflict of interest.
