# Supplementary material for: Longitudinal Analysis of COVID-19 Patients Shows Age-Associated T Cell Changes Independent of Ongoing Ill-Health
Source: Front Immunol. 2021 May 7;12:676932. doi: 10.3389/fimmu.2021.676932 (PMC8138306; doi:10.3389/fimmu.2021.676932)
Supplement: Supplementary file 1 [file DataSheet_1.pdf]

**Supplemental Figure 1: Flow cytometry gating strategy.**

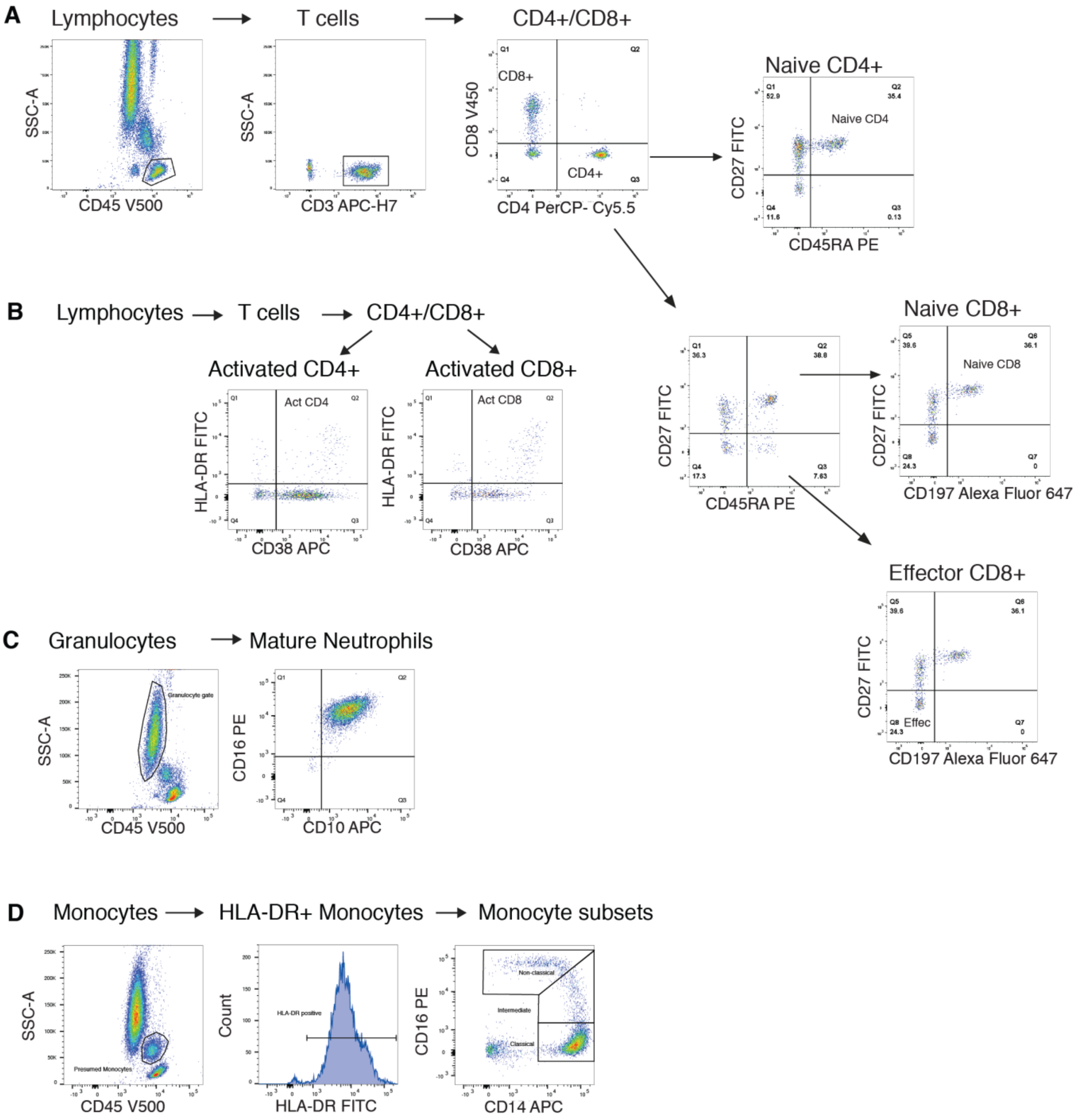


**Supplemental Figure 1:** Gating strategy for flow cytometry shown **(A)** naïve and effector T lymphocytes **(B)** activated T lymphocytes **(C)** neutrophils **(D)** monocytes

**Supplemental Figure 2: Patient enrolment diagram**

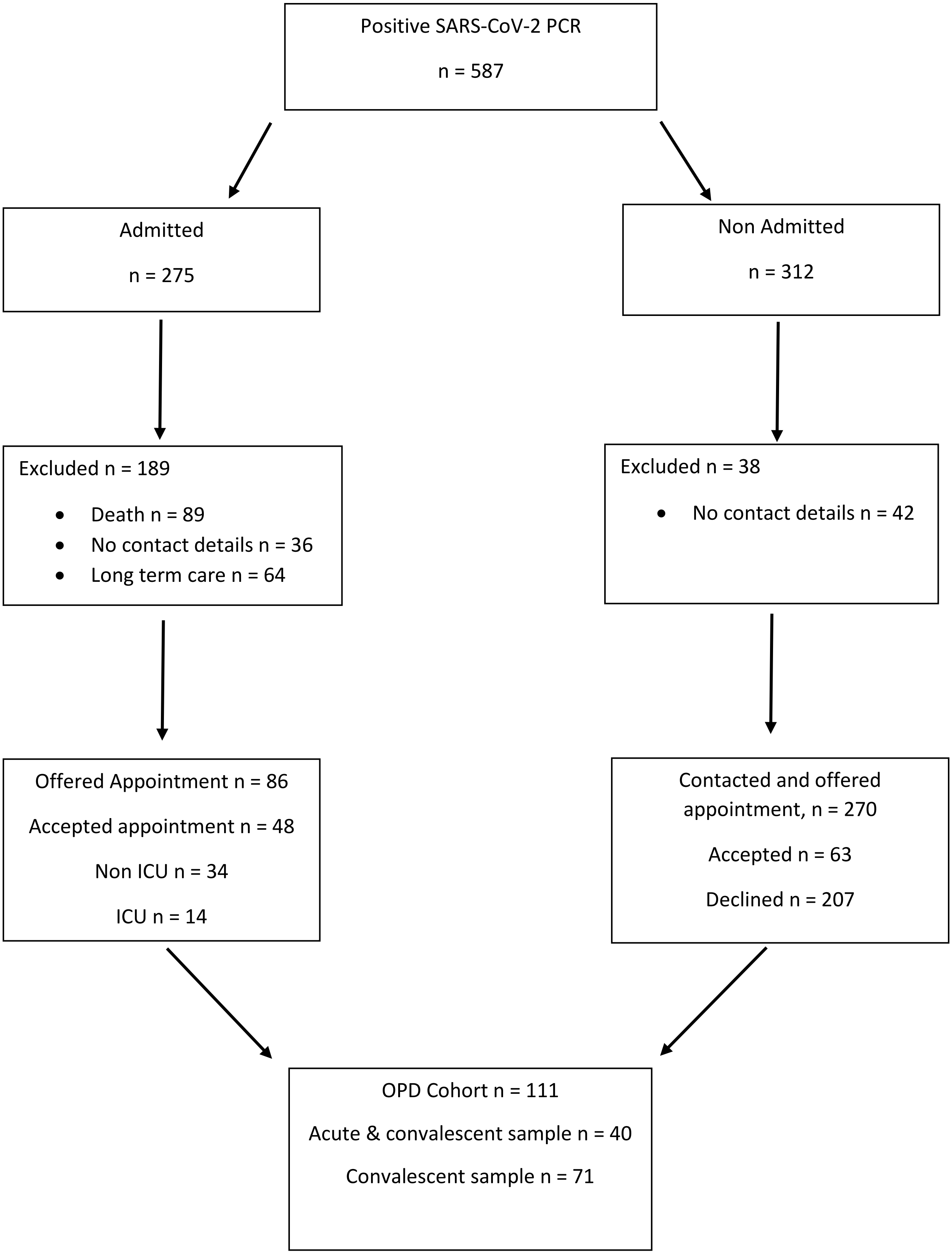

**Supplemental Figure 3: Lymphoid subsets in the matched cohort at 68 days post-infection versus controls, stratified by need for admission during acute infection**

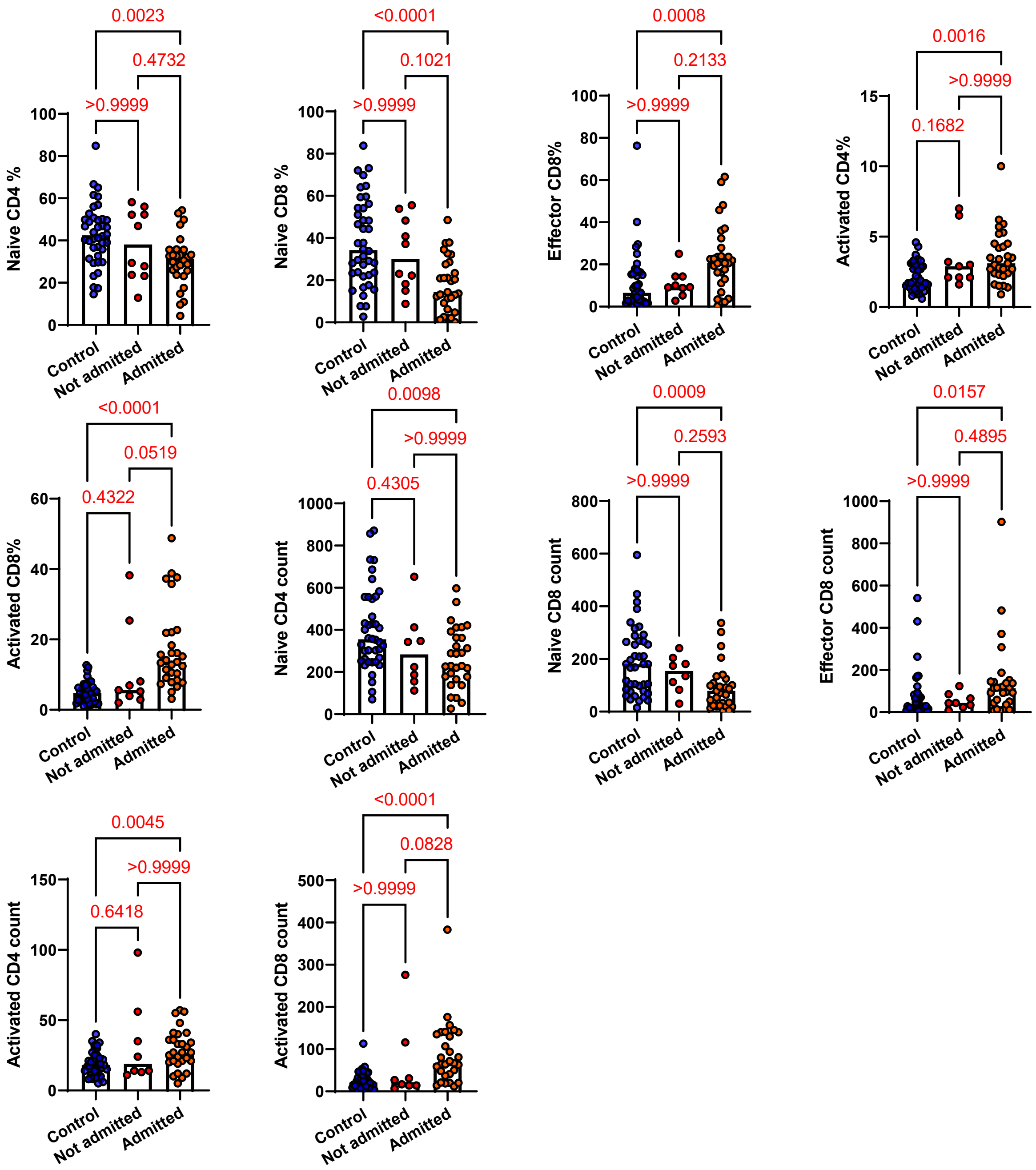


**Supplemental Figure 3:** Lymphocyte subpopulations in n=40 convalescent COVID patients, divided by need for admission during acute infection, in comparison to n=40 controls. Proportions and absolute counts of cell populations shown. Kruskal-Wallis test with Dunn's multiple comparison post-hoc test used to determine between-group differences.

**Supplemental Figure 4: Recovery of B and NK cells**

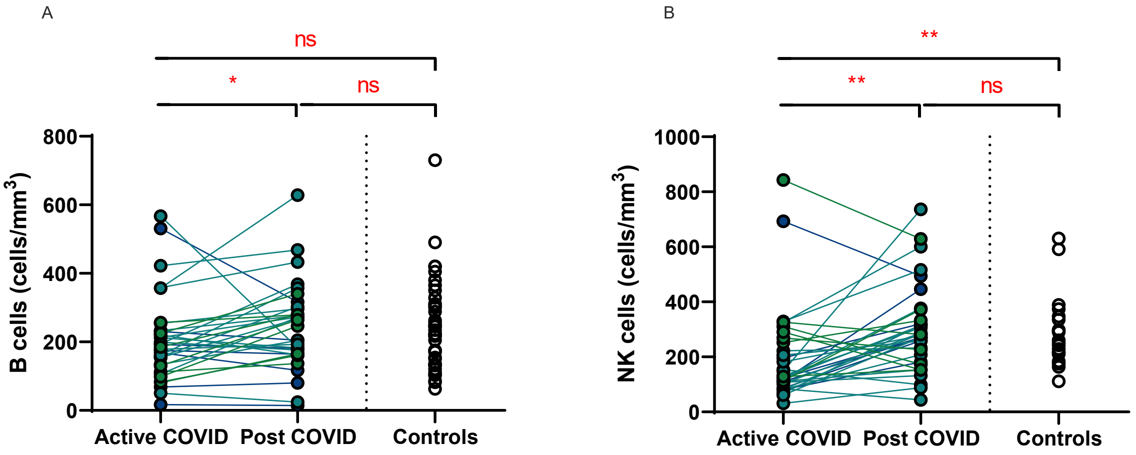


**Supplemental Figure 4:** Matched peripheral whole blood lymphoid cell counts from n=40 patients recovered from COVID-19 versus uninfected controls (n=40). **(A)** B cells **(B)** NK cells. Wilcoxon rank-sum (unpaired) and Wilcoxon sign-rank (paired) tests.* p <0.05, ** p <0.01, ns = Not Significant

**Supplemental Figure 5: Major lymphoid populations in convalescent COVID-19.**

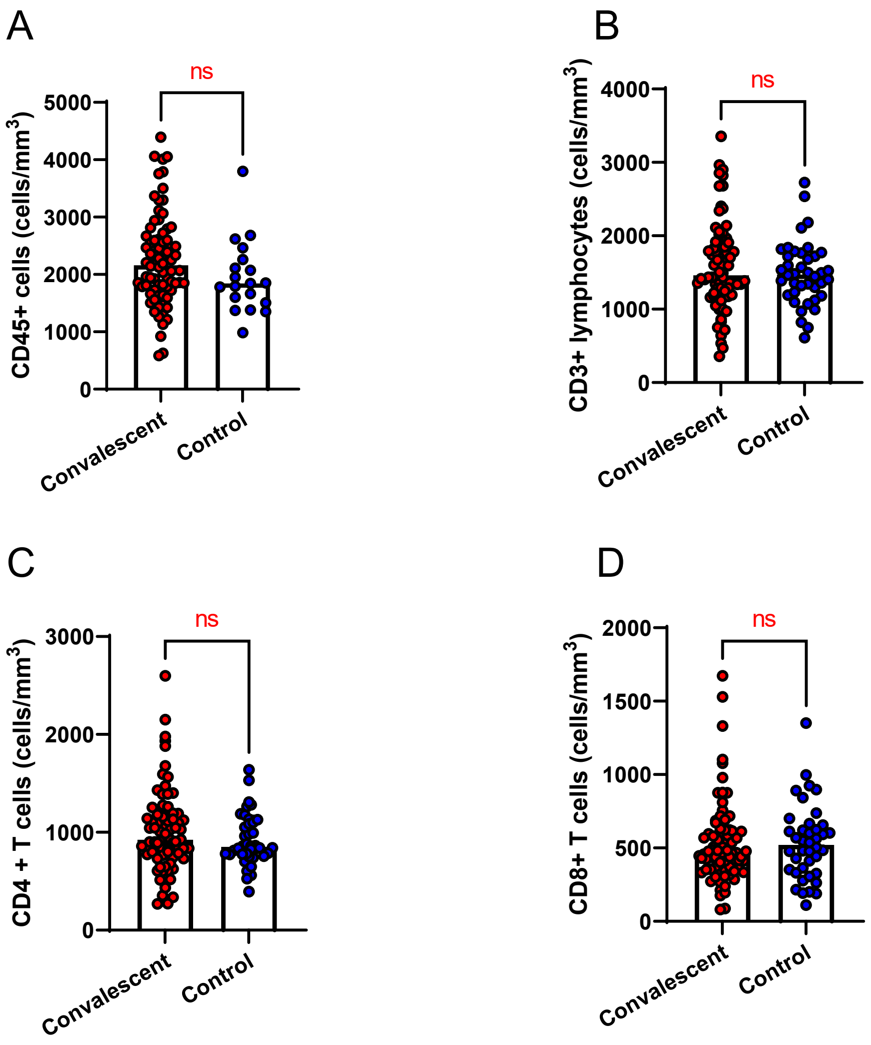


**Supplemental Figure 5:** Major lymphoid populations in n=71 convalescent COVID patients in comparison to n=40 controls **(A)** CD45+ cells **(B)** CD3+ lymphocytes **(C)** CD4+ T cells **(D)** CD8+ T cells. Wilcoxon rank-sum test ns=not significant

**Supplemental Figure 6: Lymphoid subsets at convalescence, stratified by need for admission during acute infection**

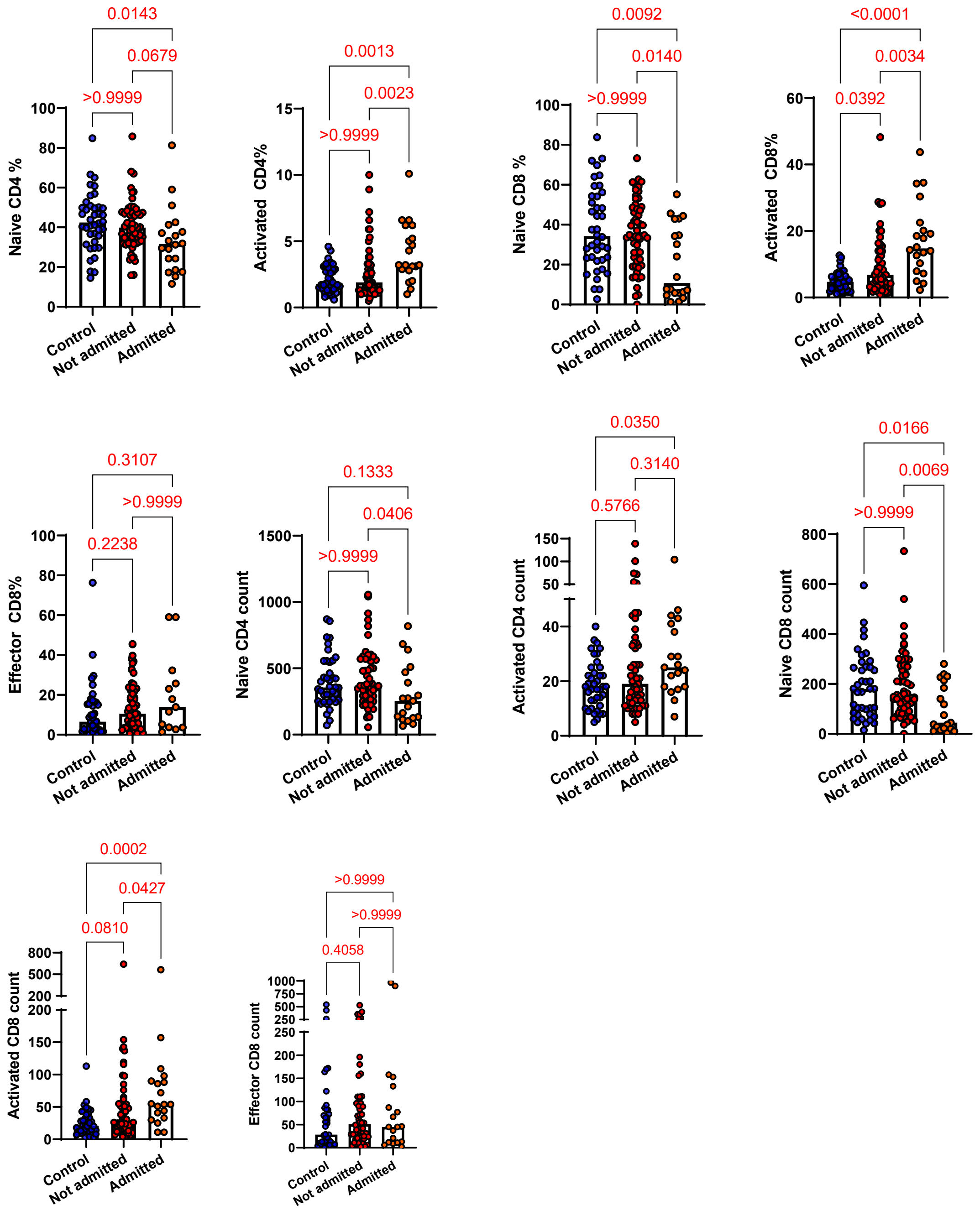


**Supplemental Figure 6:** Lymphocyte subpopulations in n=71 convalescent COVID patients, divided by need for admission during acute infection, in comparison to n=40 controls. Proportions and absolute counts of cell populations shown. Kruskal-Wallis test with Dunn's multiple comparison post-hoc test used to determine between-group differences.

**Supplemental Figure 7: Monocyte subpopulations in convalescent COVID-19.**

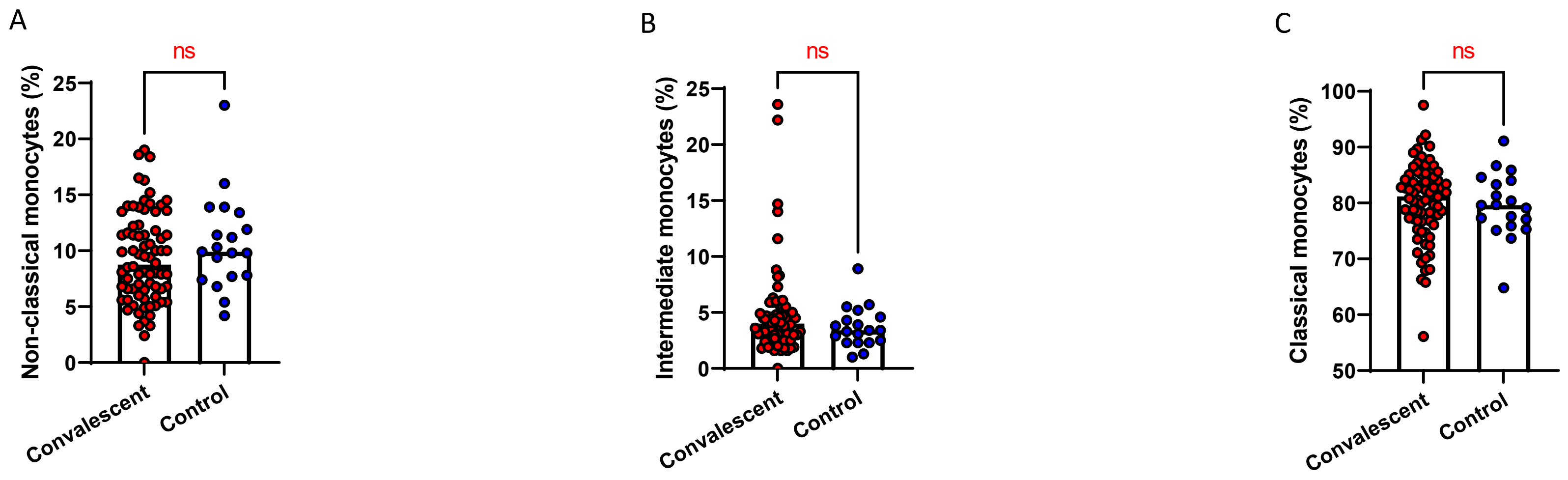


**Supplemental Figure 7:** Monocyte subpopulations in n=71 convalescent COVID patients in comparison to n=20 controls **(A)** non-classical monocytes **(B)** intermediate monocytes **(C)** classical monocytes. Wilcoxon rank-sum test. ns=not significant

**Supplemental Figure 8: Age-associated changes in convalescent lymphocyte subsets**

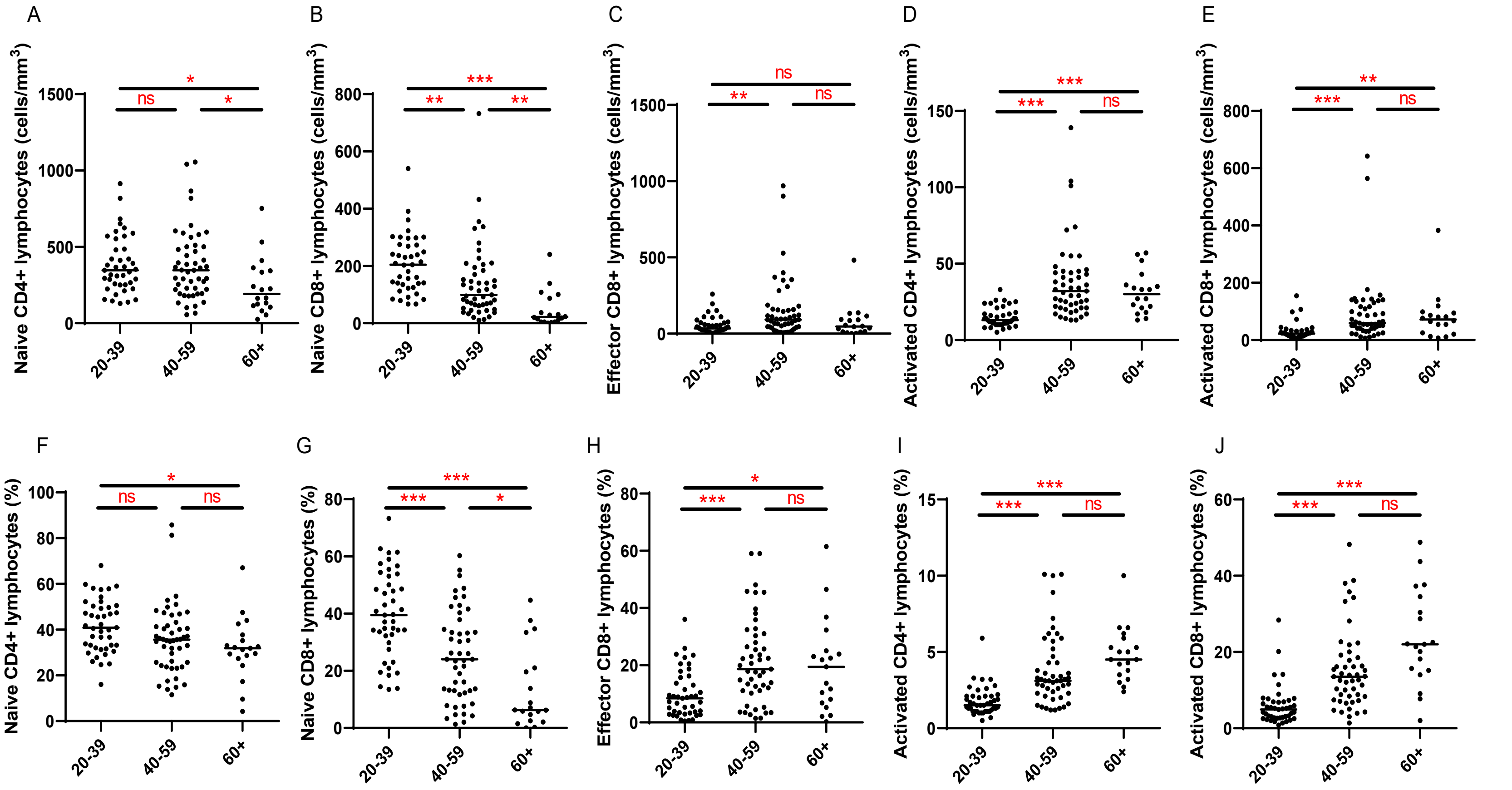


**Supplemental Figure 8:** Lymphocyte immunophenotyping of convalescent COVID patients (n=111) broken down by age. **(A)** naïve CD4+ T cell count **(B)** naïve CD8+ T cell count **(C)** effector CD8+ T cell count **(D)** activated CD4+ T cell count **(E)** activated CD8+ T cell count **(F)** naive CD4+ T cell proportion **(G)** naïve CD8+ T cell proportion **(H)** effector CD8+ T cell proportion **(I)** activated CD4+ T cell proportion **(J)** activated CD8+ T cell proportion. Kruskal-Wallis test with post-hoc Dunn test.* $p < 0.05$, ** $p < 0.01$, ***$p < 0.001$, ns = Not Significant

**Supplemental Figure 9: Age-associated changes in convalescent lymphocyte subset proportions versus age-matched controls**

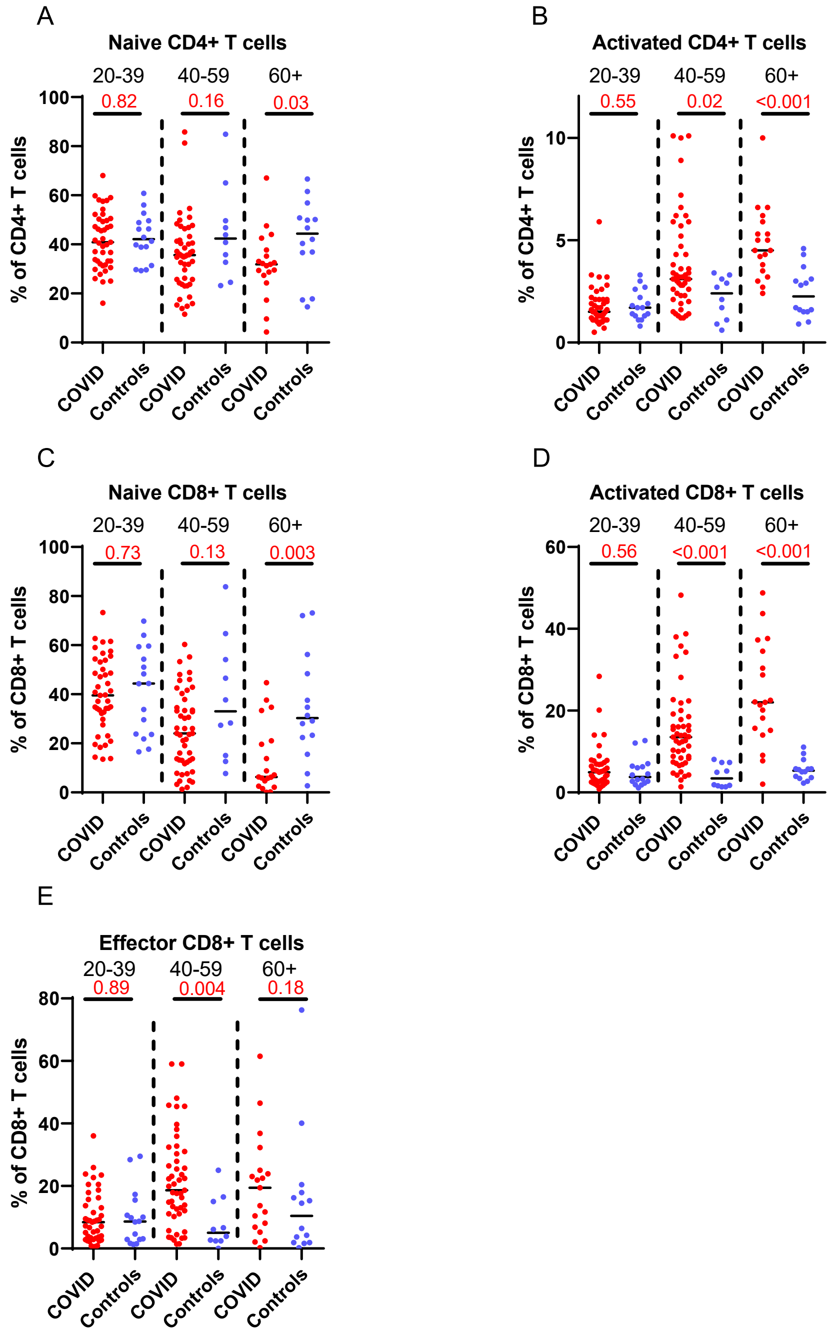


**Supplemental Figure 9:** Lymphocyte immunophenotyping of convalescent COVID patients (n=111) broken down by age with age-matched controls, sowing proportions of naïve (A) and activated (B) CD4+ T cells, and naïve (C), activated (D) and effector (E) CD8+ T cells. Differences assessed by Wilcoxon rank-sum test. Bonferroni correction, significance p<0.02

## Supplemental Figure 10: Relationships of monocyte subsets with physical health measures

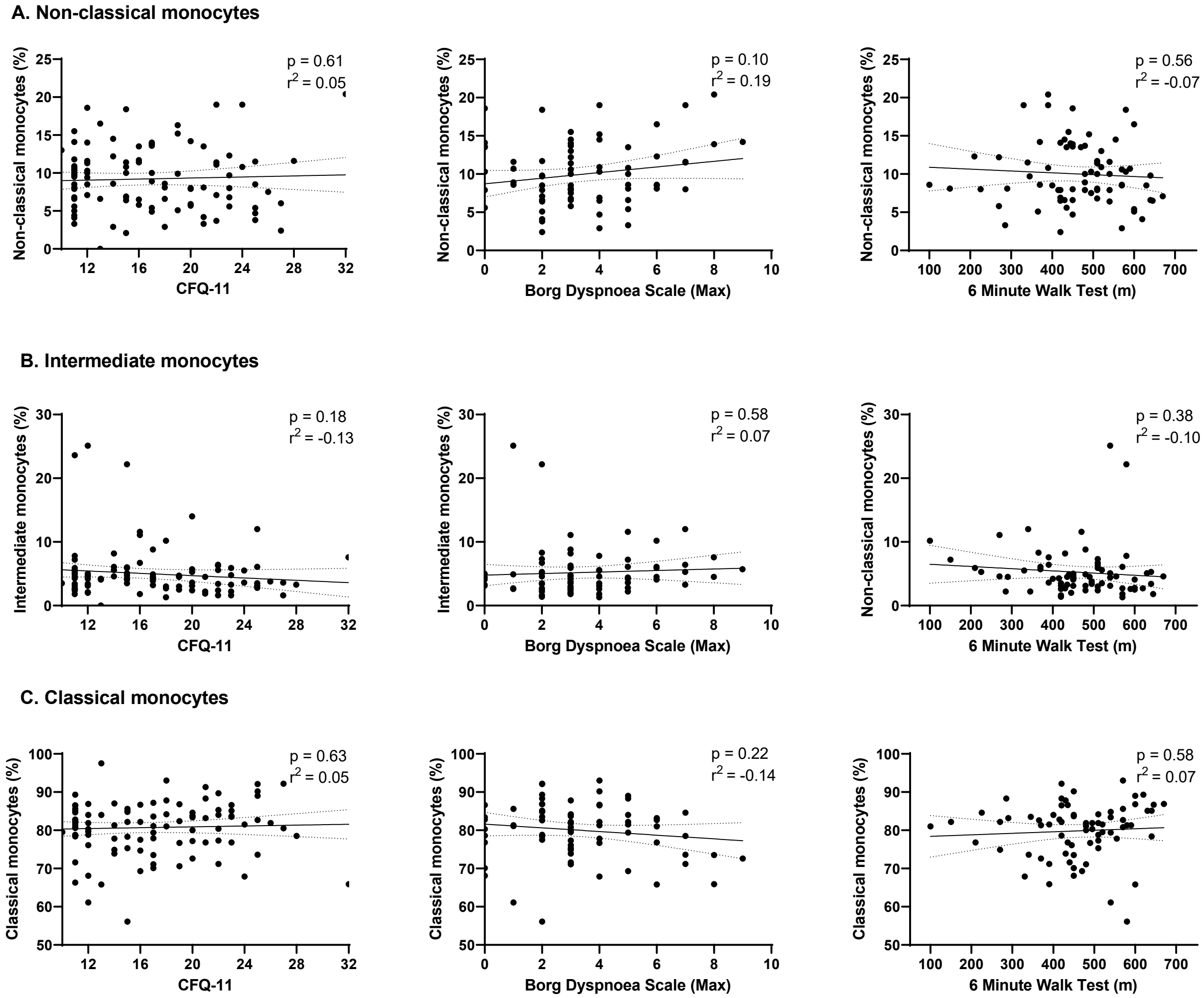


**Supplemental Figure 10:** Relationship with fatigue, perceived exertion and 6MWT distance in n=101 convalescent COVID patients with (A) non-classical monocytes (B) intermediate monocytes (C) classical monocytes. Correlation assessed with Pearson's chi-squared test. CFQ-11 = Chalder Fatigue Questionnaire-11

**Supplemental Table 1: Flow cytometry antibodies**

| Antibody | Fluorophore | Clone | Company | Catalogue # |
|---|---|---|---|---|
| CD45 | V500-C | 2D1 | BD Biosciences | 655873 |
| CD8 | V450 | RPA-T8 | BD Biosciences | 560347 |
| CD3 | APC-H7 | SK7 | BD Biosciences | 641415 |
| CD4 | PerCP/CY5.5 | Sk3 | BD Biosciences | 332772 |
| CD45RA | PE | - | BD Biosciences | 556627 |
| CD27 | FITC | - | BD Biosciences | 555440 |
| CD197 | Alexa Fluor 647 | 150503 | BD Biosciences | 560816 |
| HLA DR | FITC | L243 | BD Biosciences | 347400 |
| CD38 | APC | HB-7 | BD Biosciences | 345807 |
| CD14 | APC | MΦP9 | BD Biosciences | 345787 |
| CD16 | PE | B73.1 | BD Biosciences | 332779 |
| CD10 | APC | HI10a | BD Biosciences | 332777 |
